# Digital Inventory of Swiss Construction Systems

**DOI:** 10.1038/s41597-025-06101-6

**Published:** 2025-11-18

**Authors:** Aldrick Arceo, Maléna Bastien-Masse, Barbara Lambec, Corentin Fivet

**Affiliations:** École Polytechnique Fédérale de Lausanne (EPFL), Structural Xploration Lab, 1700 Fribourg, Switzerland

**Keywords:** Civil engineering, Databases

## Abstract

Wider implementation of circular economy practices in the construction sector requires predicting the availability, properties, and qualities of reusable components beyond mere material quantities. However, data describing the geometric attributes, material properties, and modes of assembly of products available in current buildings is limited. To address this data gap, we present the Digital Inventory for Swiss Construction Systems (DISCS), a database structure that provides detailed information on component attributes in as-built load-bearing and insulating layers of existing buildings. The dataset currently provides granular data for 102 buildings in Switzerland, each digitalised into a building information model and parameterised using a custom library of 78 attributes. The database structure facilitates the operationalisation of an inventory for construction systems, providing a basis for stock prediction that supports the upscaling of component reuse in new projects. This data descriptor motivates the need for such a database, describes its ontology, and validates its use through a series of first-level analyses.

## Background & Summary

The built environment is a potential source of construction components and secondary materials^1^. When circular economy is applied to the construction sector, building components reclaimed from existing buildings are reused in the construction of a new one or their matter is recycled for other construction applications^2^. This concept – also known as urban mining – keeps materials within a closed loop and contributes to minimising waste generation, raw material extraction, and greenhouse gas emissions associated with manufacturing^3^. However, a current barrier to the upscaling of building component reuse is a lack of knowledge, during the early phases of the design process, on what component from *donor* buildings – i.e. obsolete structures to be deconstructed – will be available during the construction phase of *receiver* buildings – i.e. new structures that will reuse components^4,5^. From the perspective of receiver building designers, a detailed – effective or predicted – inventory of available building components is expected to reduce design uncertainty and procurement risks. From the perspective of donor building owners, similar data is expected to expedite the reusability assessment of building components, including those hidden under superficial building layers, to precisely determine their capacity to match future demand, and hence to more confidently anticipate the economic viability of their careful deconstruction and storage.

Detailed inventories of construction components are typically developed using top-down modelling and bottom-up approaches^6,7^. A top-down approach estimates building and material stocks and flows based on nationwide statistics and databases^8^. In contrast, a bottom-up approach utilises a series of parameterised building archetypes to describe material quantities and characteristics^9,10^. Between the two approaches, the bottom-up approach facilitates a better understanding of the types, quantities, and properties of components at the building level^11,12^. Limitations to the development of construction component databases are attributed to the strict access to building information, such as drawings and bills of materials, as well as the resource-intensive nature of quantifying components and measuring their dimensions^13–15^.

Existing building datasets have made material intensity data available from buildings in many world regions, particularly North America, Europe, and some countries in Asia^16^, quantified material intensity ranges that represent the variability inherent in buildings^17^, and categorised the materials by functional uses and product categories using classification systems^13^. These databases support material stock and flow studies, the assessment of environmental impacts associated with building material production, and the comparison of building design and material choices. However, the granularity of these databases is limited as information on component dimensions, connections, and detailed material properties is often excluded. The granularity of information is an important feature of a dataset for operationalising component reuse, as identified by previous studies^4,18,19^.

Here, we introduce the digital inventory of Swiss construction systems (DISCS). DISCS is a comprehensive database containing detailed construction attributes of 102 buildings, with a specific focus on load-bearing components, building envelope, and interior finishes. Having a standard digital inventory of construction systems provides a database framework that contributes to upscaling construction component reuse in new projects. This supports designers in determining the type of component and their dimensions that can be extracted and reused from a building at its end-of-life (e.g., concrete floor slabs, wood or steel elements, windows). Additionally, this provides researchers with an inventory of components, sometimes referred to as a material bank, to analyse material stocks and flows, building characteristics, or construction trends.

## Methods

The development of the database follows four main steps, which include (1) developing an ontology for the digital inventory of Swiss construction systems, (2) collecting design and construction drawings of a series of buildings, (3) modelling these buildings in a 3D bespoke Building Information Model (BIM) environment, and (4) post-processing the 3D models.

### Developing an ontology for the digital inventory of Swiss construction systems

**Fig. 1:**
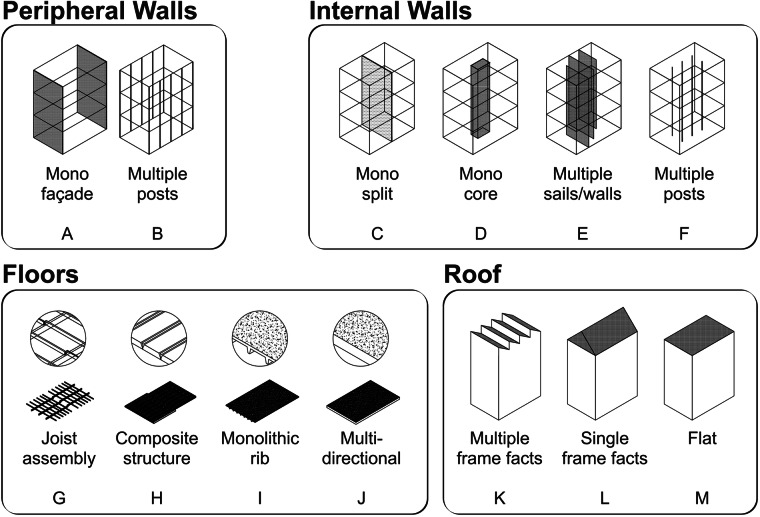
Structural typology for defining building components.

A database structure for digital inventory of Swiss construction systems was developed using a series of structural typologies (Fig. 1). The four structural typologies include external walls, internal walls, floors, and roofs, which are partly based on the construction archetypes proposed by Heisel^20^ for North America and Ostermeyer *et al*.^21^ for Switzerland. Each structural typology has two to three sub-types, each labelled by a unique letter (Fig. 1). External walls include façade-to-façade (A) and posts (B); interior walls include dividing wall (C), core (D), multiple walls (E), and posts (F); floors include primary and secondary assembly (G), structure and filling composition (H), ribbed (I), and multidirectional (J); roofs include multiple ridges (K), single ridge (L), and flat (M). Each structural typology is constructed from a variety of structural materials, which are labelled here using numbers. The common structural materials used in Swiss construction systems are cut stone (1), rubble stone (2), *in-situ* poured concrete (3), precast concrete (4), cement blocks (5), terracotta blocks (6), timber (7), and metal (8).

**Fig. 2:**
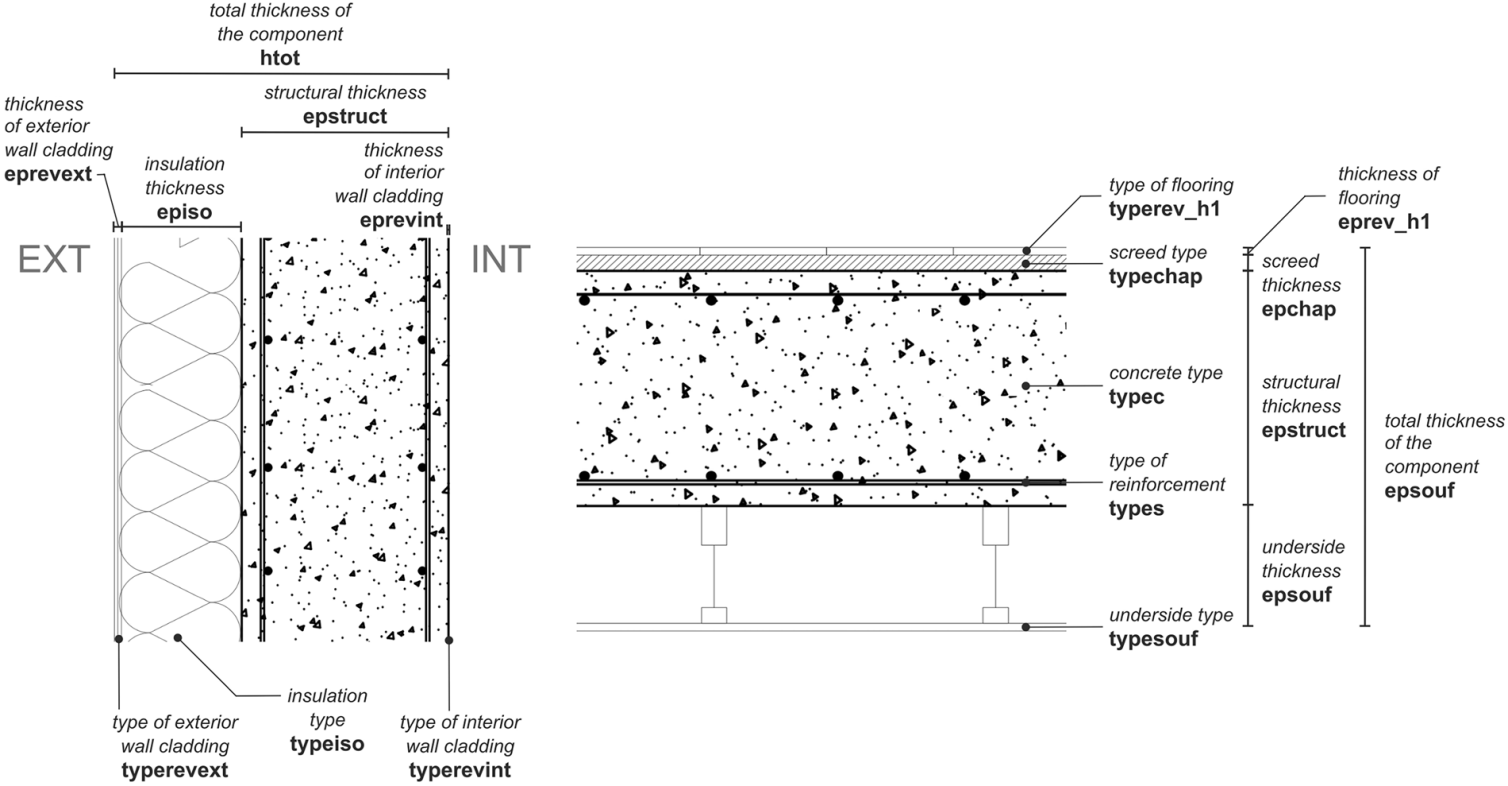
Typical parameters for wall system (DNA ‘A3’, left) and floor system (DNA ‘J3’, right).

A combination of the typology letter and material number is assigned to every building component, allowing the organisation of the elements into a “building DNA” system. For example, a floor system made of reinforced concrete flat slabs will have the DNA “J3”, while a peripheral wall made of cut stone will have the DNA “A1”. The DNA system allows the parameterisation of each component to describe its specific attributes. The attributes for the concrete floor system (J3) and concrete peripheral wall (A3) are illustrated in Fig. 2. In the concrete peripheral wall example, the attributes include the total thickness of the component (coded as htot), structural thickness (epstruct), insulation thickness (episo) and type (typeiso), exterior wall cladding thickness (eprevext) and type (typerevext), interior wall cladding thickness (eprevint) and type (typerevint). A total of 78 attributes were identified across all the buildings. The Data Records section informs on how to access information for all attributes. These attributes provide information about the structure, building envelope, or interior finishing layers of a building. The structural typology and attributes used in DISCS were defined by the authors based on their knowledge of Swiss construction and architecture, extending the parameterisation work by Ostermeyer *et al*.^21^ and general references on construction details^22^.

While structural typologies are identifiable from building drawings, their subtypes may not always be discernible. Similarly, the structural material used for these typologies may not be provided with sufficient detail. In these cases, uncategorised structural typologies were labelled using the letter “O”, and uncategorised materials were labelled with the number “0”. The occurrence of DNAs with a combination of “O” and/or “0” in the current version of the DISCS dataset is examined in the Technical Validation section. There are a few building DNAs that have two letters or two numbers. Two-lettered DNAs – e.g., “AB” or “DF” – were identified only for peripheral and interior wall components where two subtypes are present. Meanwhile, DNAs with two numbers – e.g., “37” for concrete and wood – were identified for components that combine two structural materials. Finally, there are instances where two of the same attributes were identified from the same component. For example, considering the wall system in Fig. 2, two layers of different insulation materials can be present. These were included in the dataset by having two thicknesses in the “episo” attribute and two insulation types in the “typeiso” attribute. In “episo”, the order of the presented thickness corresponds to the order of insulation types in “typeiso”.

### Collecting design drawings

Data for 320 Swiss buildings were collected in 2021 through contacts across Switzerland with large real estate owners such as pension funds, retail companies, non-profit associations, and public administrations (both cantonal and communal levels), as well as through a call for contributions from small private owners. A small number of buildings drawings were retrieved from public archives and local municipalities through building permits. All building data consist of floor plans, elevations, and cross-sections. A subset of total building data consists of 3D models, technical reports, and photos. The quality of the collected data varied between buildings and was graded using a data quality indicator (Table 1). The data quality indicator categorises the drawings into four ordinal numbers (from 1 to 4), with 4 indicating the highest quality. Categorising the drawings was based on three metrics, including the contents of the plans, their quality, and whether they have accompanying data (e.g., detailed drawing specifications). The data quality indicator was used to prioritise which buildings to model in the 3D environment first.

**Table 1:** Data quality indicators and metrics.

Score	Design drawing content	Design drawing quality	Availability of supplementary data
4	Plan views of all floors, sections, and façades are available.	Drawings are clear and readable.	Yes
3	Plans of certain floors or façades are missing.	Drawings are clear and readable.	No
2	Plans of certain floors or façades are missing, and section drawings are unavailable.	Drawings are less clear and readable.	No
1	Plans of certain floors or façades are missing, and section drawings are unavailable.	Drawings are unclear and component materials are difficult to identify.	No

Of all the collected buildings, 187 were modelled in 3D (see Modelling Buildings in 3D Environment section) after a detailed review of the drawings. The excluded buildings were missing important drawings (e.g., foundation and underground floor plans, roof cross-sections) that are necessary for extracting complete building component attributes. Of 187 buildings, 85 were later excluded due to missing details that were not identified in the initial screening. As a result, a total of 102 buildings underwent post-processing and were included in the current version of the dataset. The set comprises residential buildings, including single-family houses and multiple-dwelling buildings, as well as office, commercial, and industrial buildings. The database includes details such as municipality, year of construction, primary use, and dimensions (e.g., gross floor area, height, number of stories above and below ground) to describe each building (see Data Records).

Table 2 presents the breakdown of the 102 buildings by use category, construction year, and height, as well as the mean gross floor area for each category. The database is primarily composed of residential buildings, buildings constructed after 1946, and buildings ranging from 12 to 21 meters in height. The mean gross floor area of the 102 buildings is 2,760 m^2^, with considerable variations when examined by use categories, age cohort, and height.

**Table 2:** Count and gross floor area of DISCS buildings by total and categories.

Building categories	Number of buildings	Gross floor area (m^2^)
All	102	2,760 ± 3,040^*a*^
**Use categories**		
Single dwelling	10	235 ± 70
Multi-dwelling	75	2,450 ± 1,900
Office	11	5,190 ± 3,610
Industrial & Retail	6	6,240 ± 8,060^*a*^
**Construction period**		
Before 1919	7	1,320 ± 1,120
1919 to 1945	10	1,390 ± 1,420^*a*^
1946 to 1975	41	2,920 ± 2,030
1976 to 2000	18	2,980 ± 2,980
2001 to 2030	26	3,270 ± 4,680^*a*^
**Height**		
Above 21 m	22	4,250 ± 2,860
From 12 to 21 m	49	3,100 ± 3,480^*a*^
Below 12 m	31	1,170 ± 1,220^*a*^

^*a*^A larger standard deviation compared to mean gross floor area indicates that the building size distribution is positively skewed.

### Modelling buildings in 3D environment

The collected data were processed to model the buildings in a 3D environment using a BIM format and characterise the component attributes. The purpose of the 3D representation of the buildings is to understand the spatial distribution of the components and their connections to each other. For this reason, every load-bearing component is represented as a 2D element, informed by specific attributes that detail its surface area (i.e., BIM projections) and composition, using the ontology described in Developing an Ontology for DISCS section.

**Fig. 3:**
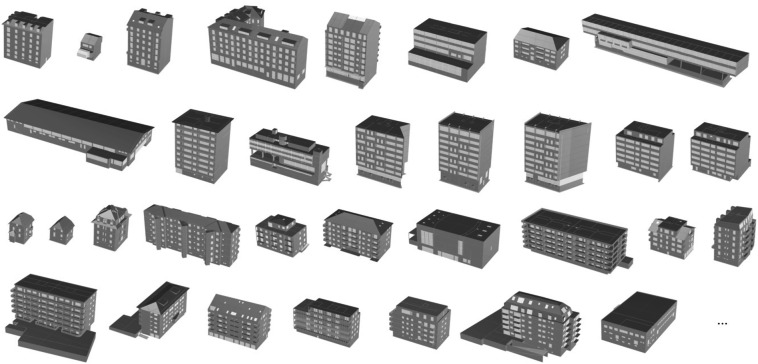
Sample 3D models of DISCS buildings.

Data available as design drawings (floor plans and elevations) were first modelled into AutoCAD to generate DWG files. Then, these files were uploaded into Rhinoceros 3D (Rhino) where the building is modelled in 3D. In this file, each modelled component is assigned to a BIM layer corresponding to its level in the building (underground levels, ground floor, common level, or roof) and a sub-layer corresponding to one of the following types: (1) peripheral components and envelope (PER), (2) opening such as windows (FEN) and doors (POR), (3) internal components (INT), (4) stairs (ESC), and (5) floor systems (PLA). Within each BIM layer, one or more components may exist, each representing a 2D element uniquely characterised by its material composition and attributes (e.g., thickness, see Developing an Ontology for DISCS section). All modelled components were then assembled to generate the 3D model of a building (Fig. 3). The 3D models adhere to the LOD300 industry modelling standard, which displays precise geometry and connections of every component^23,24^.

While component attributes were identified as being present in the buildings, there are instances when detailed data for them are not available in the dataset. For example, some buildings were identified as using a certain thickness of exterior insulation (episo), but information on the type of insulation (typeiso) was not available. Similarly, some buildings were identified as using a certain type of insulation (typeiso), but the drawings do not indicate its thickness (episo). For these instances, attributes identified in the building components but without exact information or measurement available were labelled as “missing” or “0”, respectively. The included codebook (see Data Repository) highlights that cells in the DISCS attributes with a value of zero (0) indicate a missing measurement. Attributes that were not identified in the building were left blank in the DISCS attributes dataset. This modelling choice preserves the information that can be reliably extracted from the original drawings. Guidance on interpreting and handling missing data is provided in the Usage Notes section to support confident and appropriate use of DISCS dataset. The occurrence of missing values for each attribute is described in the Technical Validation section.

Doors and windows were modelled in 3D to help in precisely measuring the dimensions of the structural components. However, these elements were not provided with attributes and have only information on their dimensions – i.e. surface area –, spatial distributions in the 3D model, and connections with other components.

### Post-processing of 3D models

The modelled buildings in Rhino were further processed in Grasshopper 3D, a Rhino plugin that enables the creation and updating of attributes. The post-processing involved discretising curves and checking for polysurfaces to enable the exporting of the components and their attributes outside of Rhino. It also involved checking whether attribute names were correct and whether attribute inputs had the right format. Grasshopper 3D was used to export the modelled buildings and their component attributes as quantity takeoffs in comma-separated value (CSV) files.

The data in these CSV files was then further curated to check for missing DNA and structural thickness (epstruct) values, as these two attributes were considered essential for the intended use of the database. Where a component had missing values for one or both attributes, the data were completed using one of the following values, in order of priority: (1) the value of connected elements of the same type (see Modelling Buildings in 3D Environment section for details of peripheral walls, interior walls, floor, and roof elements), (2) the most common value for elements of the same type in the whole building, or (3) the default values of ‘O0’ or ‘150’ for DNA and epstruct, respectively. A structural thickness of 150 mm was considered a structurally safe average for standard structural elements following Vittone^22^. Missing thicknesses for the stairs were extraplated from DISCS buildings that use the same construction type. Stairs made of stone, concrete, timber, and metal were given thicknesses of 300 mm, 200 mm, 30 mm, and 15 mm, respectively.

Finally, the material quantities of the structural components were calculated and aggregated by construction material for each building case study. The material quantities were included in the dataset and used to validate the dataset (see Validation of structural thickness and material intensity factors in the Technical Validation section).

### DISCS scope and limitations

The DISCS dataset focuses exclusively on load-bearing structural components. These elements represent the majority of a building’s material mass and account for the largest share of embodied carbon^25–27^. As such, they are often considered as the primary target for reuse strategies. While non-structural components such as windows and finishing layers also have reuse potential, their characterization can be made more easily on-site (e.g., visual inspection) and their spatial distribution is harder to predict due to higher transformation rates. For this reason, they are currently excluded from the dataset. Future development may introduce them in the same database.

The DISCS dataset maintains transparency regarding the presence of unknown DNAs (e.g., “O0”) and missing values. These gaps may affect the accuracy of reuse assessments, particularly when structural attributes are unavailable. To help mitigate these limitations, we provide indications in the Usage Notes section for users to supplement missing data.

## Data Records

The DISCS database is publicly available on Zenodo^28^. The database is stored in two CSV files named “DISCS Building Information” and “DISCS Attributes”.

- The DISCS Building Information CSV file contains pertinent information describing the geospatial and physical characteristics of the buildings. Each row represents one building in DISCS.
- The DISCS Attributes CSV file contains attributes for every component identified in the buildings in DISCS. Multiple rows are linked to a building to indicate the various components that comprise the building.

A codebook in Portable Document Format (PDF) is included to define the columns in DISCS Building Information and describe the attributes and data formatting in DISCS Attributes. It provides instructions on possible contributions to the current database. Contributions include the addition of new building case studies, building characteristics, component attributes, and material types.

## Technical Validation

### Buildings in DISCS

The DISCS dataset currently has 102 Swiss buildings of varying geographical locations, use types, and construction ages. Of the 102 buildings, 32 are located in the canton of Geneva, 28 in Vaud, 17 in Fribourg, 12 in Basel-Stadt, nine in Neuchâtel, three in Valais, and one in Bern. Approximately 88% of these buildings are located in Espace Mitteland and Lake Geneva region^29^, more specifically in the cantons of Geneva, Vaud, Fribourg, Neuchâtel, and Valais, where construction techniques are similar^30^. Considering building use types, the DISCS dataset has 10 single dwellings, 75 multi-dwellings, 11 offices, and six industrial and retail buildings. Considering five age cohorts following transition years that correspond to major changes in Swiss construction practices^31^, the DISCS dataset has seven buildings from the “before 1919” cohort, 10 from the “1919–1945” cohort, 41 from the “1946–1975” cohort, 18 from the “1976–2000” cohort, and 26 from the “2001–2030” cohort. Clustering the use types and age cohorts together (Table 3) reveals that multi-dwellings and buildings constructed after 1946 are well-represented in the DISCS dataset. This clustering also suggests that more data are needed for single-dwellings, offices, industrial, and retail buildings constructed before 1946.

**Table 3:** Cluster of buildings in DISCS dataset by use type and age cohort.

Use type	Age cohort				
	Before 1919	1919–1945	1946–1975	1976–2000	2001–2030
Single dwelling	0	3	0	2	5
Multi-dwelling	7	7	33	10	18
Office	0	0	5	5	1
Industrial & Retail	0	0	3	1	2

The data collected limit the geographical and temporal representativeness of DISCS to buildings located in Espace Mitteland and Lake Geneva regions^29^ and constructed after 1945. Future expansion of the dataset will involve treating the 85 remaining unprocessed buildings (see Collecting design drawings) and acquiring new design drawings – particularly of buildings in other Swiss regions (Northwestern Switzerland, Zurich, Eastern Switzerland, Central Switzerland, and Ticino) and of those constructed before 1945 - from large real estate owners and small private owners. Given the detailed structure and reproducibility of DISCS database, contributions from architects, engineers, and researchers are encouraged to support its expansion. See Usage Notes for guidance on contributing data.

**Fig. 4:**
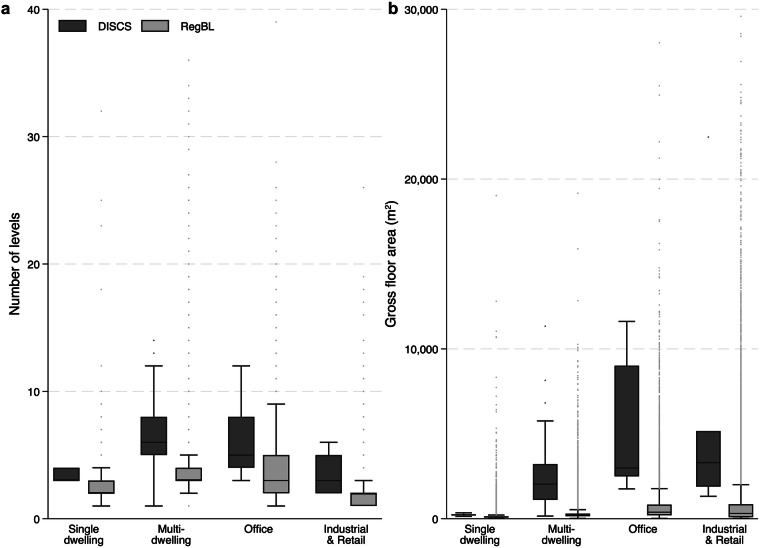
Number of levels (**a**) and gross floor area (**b**) of DISCS buildings and the Swiss building stock (RegBL) broken down by use types. The y-axis for (**a,b**) are restricted to 40 floors and 30,000 m^2^, respectively, to clearly show the interquartile ranges of the box plots.

The number of levels and gross floor area of the buildings in DISCS dataset were plotted against the Registre Fédéral des Bâtiments et des Logiments (RegBL) dataset^32^ (Fig. 4). RegBL is developed by the Swiss Federal Statistical Office and provides a reference information system for all buildings in Switzerland. It is often used for data exploration or statistical analysis on the Swiss building stock. In Fig. 4a., the DISCS dataset has median gross floor areas 2 to 11 times higher than that of the RegBL dataset across the four use types. Similarly, the DISCS dataset has a median number of floors 1.5 to 2 times higher than the RegBL dataset across the four use types (Fig. 4b). This indicates that the size and height of the buildings collected were slightly bigger than the norm. Nevertheless, the size and height of DISCS buildings are within the range of the Swiss building stock.

### Quality of design drawings

The aggregated mean data quality indicators (as described in Table 1) for the 102 buildings in the DISCS dataset, categorised by total, use type, and age cohort, are shown in Table 4. The mean data quality of all buildings is 3.3, indicating good quality data sources. When disaggregated by use type, the mean scores range from 3.1 for industrial and retail buildings to 3.6 for office buildings. Mean data quality, considering age cohorts, shows that cohorts constructed after 1919 have scores greater than 3, while those constructed before have scores below 3. The lower scores for buildings in the older cohorts are due to most collected drawings lacking sufficient detailed information and being in a file format that has less visible labels and building details.

**Table 4:** Data quality scores for the 102 DISCS buildings, by categories.

Building categories	Number of buildings	Data quality score
All	102	3.3 ± 0.8
**Use type**		
Single dwelling	10	3.2 ± 0.6
Multi-dwelling	75	3.3 ± 0.9
Office	11	3.6 ± 0.5
Industrial & Retail	6	3.3 ± 0.5
**Age cohort**		
Before 1919	7	2.7 ± 1.3
1919–1945	10	3.1 ± 0.8
1946–1975	41	3.3 ± 0.7
1976–2000	18	3.6 ± 0.8
2001–2030	26	3.4 ± 0.7

### Analysis of DISCS dataset

**Fig. 5:**
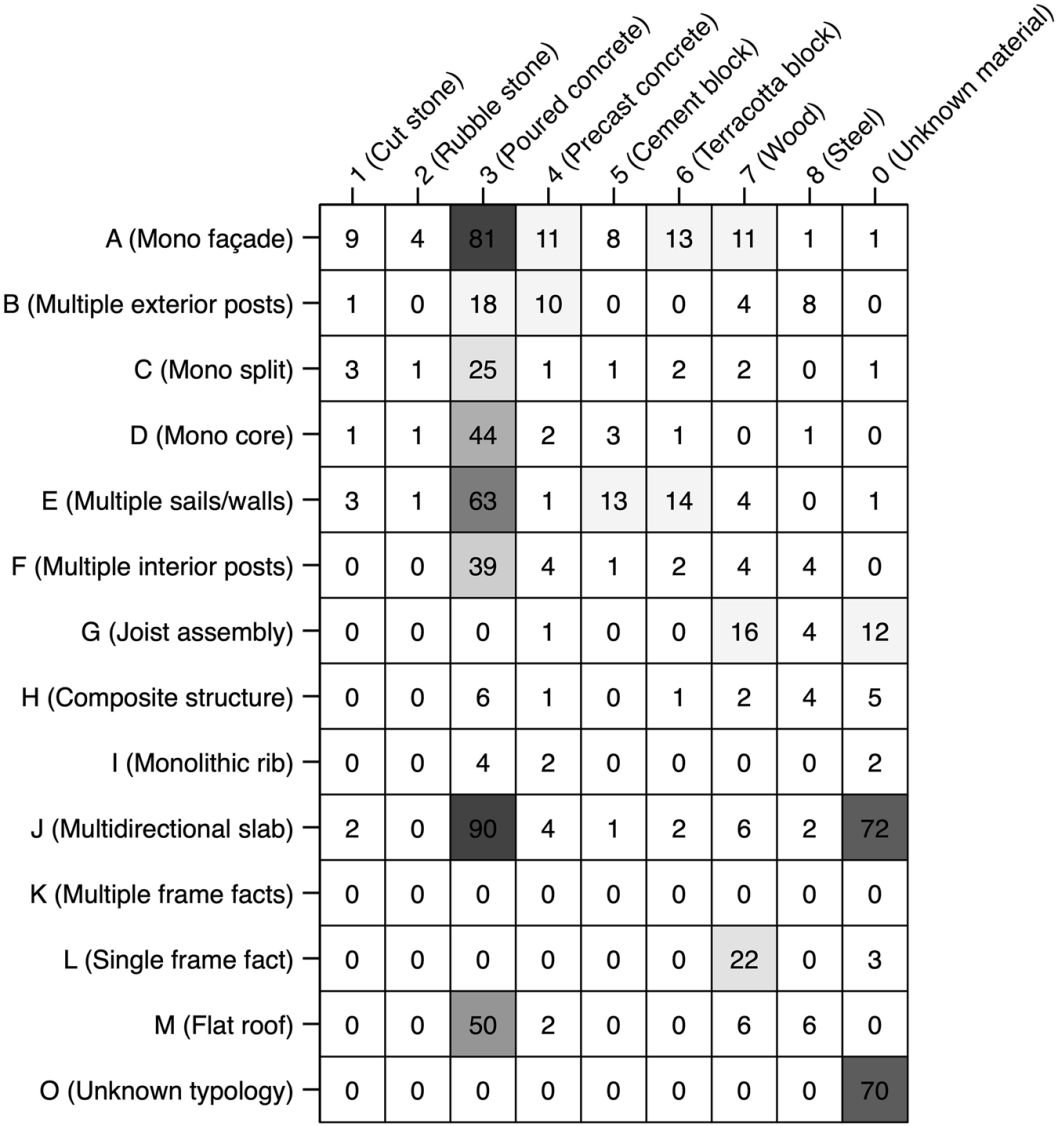
Number of building case studies in DISCS dataset that were identified to have DNA combinations. The cell contains the number of buildings that were identified to have the DNA. Each row does not add up to 102, as some buildings were identified to have two types of structural material for the same type of component.

The total number of building case studies that were identified to have specific DNA combinations were analysed to determine the quality of component identification of the DISCS buildings (Fig. 5). As indicated in the rows of Fig. 5, across the 102 buildings, the most common peripheral wall type is A (mono façade), interior wall type is D (multiple sails/walls), floor type is J (multidirectional slab), and roof type is M (flat roof). Concrete (poured on-site or precast) is the most common structural material and is commonly used in all component types. The column labelled 0 (unknown material) indicates that the materials used for some components were not identified in the drawings. For example, the type of materials for some multidirectional slabs in 72 buildings was not identified. Finally, the row labelled ‘O’ (unknown layer) indicates that the specific component type used for some components in the building was not identified – i.e., 70 buildings have components labelled as O. This was due to some drawings not having sufficient information to help identify whether a peripheral wall is constructed with a mono façade or multiple posts, an interior wall with a mono split, mono core, multiple sails/walls, or multiple posts, a floor with joist assembly, composite, monolithic rib, or multidirectional slab, and a roof with single or multiple frame facts.

To examine the magnitude of structural typology and material identification for each building, a metric was developed to estimate the DNA identification score. The DNA identification score is calculated as the ratio of component surface area in a building that was identified with non-O or non-0 DNA and total surface area (Eq. 1). The score has a value between 0–100% with a value of 100% indicating that all components in a building were identified to have DNAs without combinations of O or 0. The choice of surface area to quantify this metric is primarily due to the fact that all identified components in the DISCS dataset have this dimension measured. Unlike component mass or volume, surface area is not influenced by the type or density of material used in the components.

1 $${\rm{DNA\; identification\; score}}=\frac{\sum {{SA}}_{{with\; DNA}}}{\sum {SA}}\times 100$$

Where SA denotes surface area.

Table 5 presents the aggregated mean DNA identification scores for the 102 buildings in the DISCS dataset, categorised by total, use type, and age cohort. The mean score of all buildings is 89%. The scores of the four use types are within the range of the total mean score. Considering the scores by age cohort, buildings constructed after 1946 have mean scores greater than 90%, while those constructed before 1946 have mean scores between 70% and 80%. This trend is similar to the data quality scores – see Quality of Design Drawings section –, suggesting that older cohorts with drawings that lack sufficient detail likely contributed to their unidentified DNAs.

**Table 5:** DNA identification score for the 102 DISCS buildings, by categories.

Building categories	Number of buildings	DNA identification score (%)
All	102	89 ± 19
**Use type**		
Single dwelling	10	81 ± 29
Multi-dwelling	75	91 ± 15
Office	11	88 ± 25
Industrial & Retail	6	79 ± 34
**Age cohort**		
Before 1919	7	75 ± 28
1919–1945	10	80 ± 29
1946–1975	41	92 ± 12
1976–2000	18	90 ± 11
2001–2030	26	90 ± 23

The total number of components in the DISCS Attribute dataset is 48,779, with a mean number of 478 for each building. Considering building use types, office buildings have the most components – with a mean of 764 – followed by multi-dwellings (502), industrial and retail buildings (332), and single dwellings (73). The most common DISCS attributes were analysed by counting the number of buildings within which these attributes were identified (Table 6). The most common parameters in the database are the DNA, surface area of the components (area), the thickness of structural components (epstruct), and the thickness of total components (htot), mainly due to the curation process described in Post-processing of 3D Models section. These were identified in all 102 buildings, encompassing every structural building component, excluding windows and doors. The next most common parameters, identified in at least half of the building entries, are shown in Table 6.

**Table 6:** Most frequent attributes in DISCS dataset and mean percentage of missing value occurring.

Attribute	Description	Number of buildings with attribute	Mean percentage of missing attribute (%)^*a*^
dna	Combination of a letter for structural typology and a number for material type	102	0
area	Surface area of the component	102	0
epstruct	Thickness of structural component	102	0
htot	Total thickness of the component including non-structural layers	102	0
typetoit	Roof type	102	33
types	Type of reinforcement	97	96
typec	Type of concrete	96	85
eprev_h1	Thickness of flooring or roofing finish	92	51
types_inf_p1min	Reinforcement area of lower slab reinforcement at the same direction as the span	91	93
types_inf_p1max	Reinforcement area of lower slab reinforcement at the opposite direction of the span	91	93
types_sup_p1min	Reinforcement area of upper slab reinforcement at the same direction as the span	91	95
types_sup_p1max	Reinforcement area of upper slab reinforcement at the opposite direction of the span	91	96
as_exth	Horizontal reinforcement area of the exterior side of peripheral walls	83	94
as_extv	Vertical reinforcement area of the exterior side of peripheral walls	83	94
as_inth	Horizontal reinforcement area of the interior side of peripheral walls	83	94
as_intv	Vertical reinforcement area of the interior side of peripheral walls	83	95
typerevext	Type of exterior wall cladding	79	4
eprevext	Thickness of exterior wall cladding	79	48
typerev_h1	Type of flooring finish	75	35
episo	Insulation thickness	70	9
typeiso	Type of insulation	70	42
typerevint	Type of interior wall finish	54	17
eprevint	Thickness of interior wall finish	54	36

^*a*^Percentage of missing attribute for each building, calculated as the ratio of the surface area with missing value and total surface area of components where the attribute was identified. The mean value was calculated for all buildings with the identified attribute.

In developing the DISCS dataset, attributes were only entered when the data collected allowed their identification with sufficient confidence. Consequently, some attributes in column 3 of Table 6 are less than 102 because some buildings were not identified with the attribute. For example, some concrete buildings do not use exterior cladding, which results in the dataset having no input for eprevext (thickness of exterior wall cladding) and typerevext (type of exterior wall cladding) for these buildings.

In Table 6, column 4 quantifies the mean percentage of missing values occurring across all buildings where the attribute was identified. From the subset of total attributes in the DISCS dataset, the four most frequent attributes – i.e., dna, area, epstruct, htot – have no missing values. The attributes related to concrete reinforcement – e.g., types, types_sup_p1max, as_intv – have a percentage of missing values above 90%. This high percentage is due to the design drawings not specifying this information, despite reinforcement being identified in both cast-in-place and precast concrete components.

### Validation of structural thickness and material intensity factors

The dataset was validated by comparing the thicknesses of structural components and material intensity factors with those of existing datasets from Swiss buildings.

**Fig. 6:**
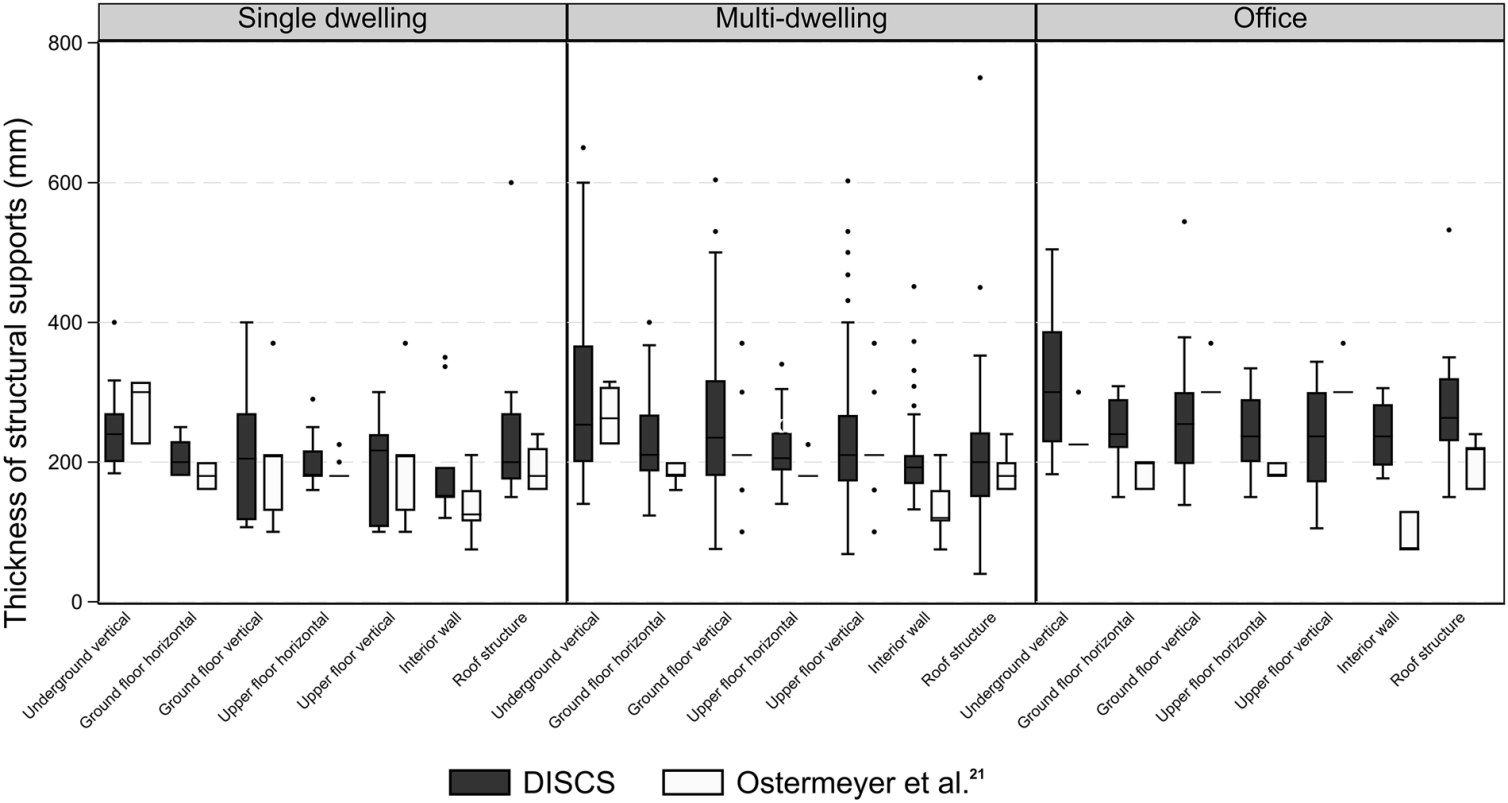
Thicknesses of structural components in DISCS and Ostermeyer *et al*.^21^ datasets by use type.

Figure 6 shows a comparison of structural thicknesses between DISCS buildings and the Ostermeyer *et al*.^21^ dataset across three building use types. The structural components are broken down across seven construction groups (underground vertical supports, ground floor horizontal supports, ground floor vertical supports, upper floor horizontal supports, interior walls, and roof structure). Across construction groups, the thicknesses of structural supports for single dwellings and multi-dwellings in the DISCS dataset are within the ranges reported in the Ostermeyer *et al*.^21^ dataset. However, DISCS notably has a wider variance and slightly thicker structural supports on average. In contrast, the thicknesses of structural supports for office buildings in the DISCS dataset are considerably larger than the buildings in the Ostermeyer *et al*.^21^ dataset. The wider variance and slightly thicker structural supports in the DISCS dataset are likely due to its incorporation of a variety of real-world building examples, which reveals heterogeneity in the way buildings are designed^27^. On the contrary, the thicknesses in construction systems provided by Ostermeyer *et al*.^21^ were based on typical construction practices, which likely provide the minimum required dimensions to meet local or national building code requirements.

Table 7 presents the material intensity factors for the DISCS buildings and building case studies from three Swiss studies. When the DISCS dataset is compared to the study by Fivet *et al*.^31^, the material intensity factors are, on average, 32–43% higher for single dwellings, 20–42% higher for multi-dwellings (except for the 1946–1975 cohort), 2–59% higher for office buildings (except for the 1946–1975 cohort), and 47–104% higher for industrial and retail buildings. However, it can be noted that material intensity factors between the two datasets are within one standard deviation of each other for some use type and age cohort – e.g., multi-dwellings constructed before 1919. Comparing the DISCS buildings with those from Heeren and Fishman^16^ and Moisteiro-Romero *et al*.^33^, the material intensity factors are 15–49% higher. As the data points compared here represent different Swiss buildings, the observed discrepancies can be attributed to variations in the construction methods of buildings of similar typologies, which consequently affect the material intensity factors. The observed variability in material intensity has been found in previous studies that examined material intensity factors between buildings of similar typology in Canada, Australia, and the Philippines^14,34,35^.

**Table 7:** Material intensity factors from other previous material stock studies in Switzerland and the DISCS dataset.

Source	Use type	Age cohort	Total material intensities^*a*^		Unit
			Source	DISCS	
Fivet *et al*.^31^	Single dwelling	Before 1919	0.61 ± 0.08		m^3^/m^2^
		1919–1945	0.6 ± 0.01	0.86 ± 0.2	
		1946–1975	0.61		
		1975–2000	0.5 ± 0.09	0.66 ± 0.01	
		After 2000	0.55 ± 0.08	0.76 ± 0.1	
	Multi-dwelling	Before 1919	0.61 ± 0.05	0.76 ± 0.2	
		1919–1945	0.5 ± 0.03	0.6 ± 0.2	
		1946–1975	0.51 ± 0.06	0.43 ± 0.2	
		1975–2000	0.43 ± 0.05	0.56 ± 0.1	
		After 2000	0.43 ± 0.01	0.61 ± 0.1	
	Office	Before 1919	0.66		
		1919–1945	0.59		
		1946–1975	0.58 ± 0.15	0.54 ± 0.1	
		1975–2000	0.39	0.62 ± 0.2	
		After 2000	0.47 ± 0.01	0.48	
	Industrial and retail	Before 1919	0.82		
		1919–1945	0.29		
		1946–1975	0.43 ± 0.01	0.77 ± 0.2	
		1975–2000	0.57 ± 0.35	0.84	
		After 2000	0.54 ± 0.07	1.1 ± 0.4	
Heeren and Fishman^16^	Residential buildings	All age cohorts	910	1046 ± 440	kg/m^2^
Mosteiro-Romero *et al*.^33^	Single dwelling	2011–2020	901	1343	kg/m^2^

^a^The material intensities of the DISCS buildings were adjusted to match the units and age cohorts of the other studies.

The validation of the DISCS dataset and its observed variability provide valuable insights into design uncertainty and reuse feasibility. For example, the detailed information on component thickness and dimensions (e.g., span and width) provides designers with more realistic expectations about available components, thereby reducing uncertainty when designing with reused components^36^.

## Usage Notes

The two DISCS databases are provided as CSV files, which can be read by conventional analysis software (e.g., Microsoft Excel, MATLAB, R). Special attention must be given to using the DISCS attributes file in relation to (1) DNAs with unknown structural typologies and/or materials, such as “O0”, (2) missing values - i.e. a distinction between “0” cells and empty cells or between “missing” and empty cells -, and (3) attributes with two or more observations. These are described in the Methods section. To address unknown DNAs, users are advised to extrapolate the structural typology, material, or both from other DISCS buildings with similar use types, construction periods, and locations. For missing values concerning structural components, users should consult relevant building codes and standards provided by the Swiss Society of Engineers and Architects (SIA), such as the standard for existing structures^37^. Missing data related to building envelopes and finishes can be extrapolated from comparable DISCS buildings.

Data contributions can be in the form of new Swiss building case studies, additional building DNA, or new attributes. Contributions are made by sending the data to the Structural Xploration Lab (SXL) at EPFL (see Codebook in Zenodo^28^). For new building additions, contributors should follow the attributes and format provided in the codebook to maintain consistency. For any new DNA or attribute suggestions, contributors should simultaneously update the DISCS attributes CSV files and codebook to ensure that any additions to the database structure are directly reflected in both files. The database maintainers will review any suggested update and addition to DISCS.

## Data Availability

The dataset is openly available at https://zenodo.org/records/17098001.

## Code availability

The development of the DISCS dataset does not use any custom codes.
